# The Swine Flu Episode and the Fog of Epidemics^1^

**DOI:** 10.3201/eid1201.051132

**Published:** 2006-01

**Authors:** Richard Krause

**Affiliations:** * National Institutes of Health, Bethesda, Maryland, USA

**Keywords:** influenza, swine flu, pandemics

## Abstract

The 1918 influenza pandemic has shaped research and public health for nearly a century. In 1976, the specter of 1918 loomed large when a pandemic threatened the country again. Public health officials initiated a mass vaccination campaign, but the anticipated pandemic failed to occur. An examination of the available data in 1976 and the decision to vaccinate, as well as lessons learned from the HIV/AIDS epidemic in the early 1980s, may help shape an appropriate public health response to future threats from avian influenza or other infectious diseases.

## Abstract 2

Knowledge of the 1976 swine flu episode can help shape public health response to future pandemic threats.

"Maye it please your Honor immediately upon the Queen's arrival here, she fell acquainted with a new disease that is common in this town, called here the *Newe Acquayantance*, which passed also throughe her whole Courte, neither sparing lordes, ladies nor damoysells, not so much as either Frenche or English… There was no appearance of danger, nor manie that die of the disease, excepte some olde folks. I am ashamed to say that I have byne free of it, seeing it seketh acquayantance at all man's handes."

**—Written in a letter in 1562 by Sir Thomas Randolph, ambassador from Queen Elizabeth I to the court of Mary, Queen of Scots, Edinburgh, to Cecil in London** (*1*).

I read the 1953 lecture on Influenza: the Newe Acquayantance (*1*) by Thomas Francis, Jr, in 1953, and I did not read it again until recently, as I was preparing this article. On reflection, I wish I had reread it during the swine flu episode in 1976. Certainly Francis's lecture, and his conclusions and speculations about the mysteries of 1918 influenza, should temper our strategies for coping with a possible human pandemic arising, like a phoenix, from the current influenza epidemic in Asian chickens.

In light of an influenza outbreak at Fort Dix, New Jersey, in February of 1976, the Public Health Service decided to prepare an influenza vaccine with the Fort Dix strain and immunize a large segment of the US population. Mass immunization was achieved by October of that year, although the predicted pandemic never occurred.

Now, 30 years later, we are faced with the threat of an influenza pandemic that might emerge from a massive outbreak of avian influenza H5N1 in Asian chickens. Many scientists and public health professionals who must now make decisions about the public health response are not virologists or influenza experts, as I was not, and they will need to base their decisions on expert opinion and their own evaluation of the facts. In 1976, I supported the decision to begin mass immunization, and this article examines the data and experiences that contributed to that decision. I hope my reflections will be useful for those who must determine the public health response to the threat of H5N1 in 2005. They have my best wishes.

## Early Experiences with Influenza

At the beginning of the 20th century, the fact that many contagious diseases were caused by microbes was well established, but at the time no treatment was available for any of them except syphilis and malaria. Anxiety and alarm were widespread among those who lived through the devastating 1918 influenza pandemic about the potential for a recurrence. In 1918, my parents and my brothers, then children, were living in a small town in southeastern Ohio. When I was a teenager in the 1930s, I recall my mother's reflections on the influenza pandemic. Our home at the time was near a chair factory, and after work many of the employees walked past our house. Occasionally, a worker would spit phlegm or tobacco on the pavement. For such occurrences, my mother always had a kettle of boiling water ready, so she could immediately scald the "damned spot," hoping to kill the unseen germs and protect my brothers from influenza.

I relate this anecdote as a reminder that as recently as the 1930s, when I was a teenager, the 1918 pandemic was a living memory. To this day, that pandemic casts the longest shadow, although the AIDS pandemic will likely take its place.

My next experience with influenza was in 1944, when I was in the US Army. The influenza vaccine had just been developed by Francis, Jonas Salk, and others. Their work had been supported by the army under the auspices of the Armed Forces Epidemiology Board (AFEB), for whom the pandemic of 1918 was still fresh: 50,000 soldiers had died of influenza. We GIs were lined up at the dispensary and given the vaccine, one soldier after another, with the same 50-mL syringe.

To this day, I recall the moderately severe local reaction, swelling, considerable tenderness, and pain at the injection site, and many soldiers had systemic reactions. I remember that the vaccine in the syringe was turbid, but did not know at the time that it had been grown in eggs. I have wondered since then if the turbidity of the vaccine was due to a residue of chicken feathers! Clearly, purification had a long way to go in 1944.

## Swine Flu

From 1970 to 1974, I was a member of the National Institute of Allergy and Infectious Diseases (NIAID) Infectious Disease Advisory Committee. Several times a year, we reviewed various protocols for evaluating vaccines, including influenza, that were conducted in the vaccine evaluation units then supported by NIAID. We were kept abreast of the efforts to match the influenza virus strains incorporated into the vaccines with the anticipated wild strains that would circulate in the coming season.

In the first months of 1976, mere weeks after I had become director of NIAID, influenza broke out at Fort Dix, New Jersey. Several soldiers died, and soon the Center for Disease Control (CDC) and other agencies determined that the cause was a swine flu virus (H1N1), thought to be a direct descendant of the virus that caused the pandemic of 1918. This conclusion was based on antibodies to H1N1 antigens found in survivors of the 1918 pandemic, and the belief that the 1918 virus was eventually transmitted to pigs in the Midwest, where it persisted and caused sporadic human cases. Had the virus broken out of the pigsty, so to speak, and caused the outbreak in humans at Fort Dix?

Approximately 200 young men were infected in January and February, as detected by conversion of serial sera from negative to positive for swine flu hemagglutinins. This finding was reported by Frank Top to the AFEB. With the exception of 1 or 2 deaths, the disease was reported to be mild.

Sometime in February 1976 a group of intramural and extramural influenza experts reached a near consensus that the Fort Dix swine flu was likely to be the source of an imminent pandemic of influenza, perhaps similar to the pandemic of 1918, because Fort Dix virus had the antigenic characteristics of what was thought to be the 1918 virus. One notable exception to this consensus thought it possible but unlikely that the Fort Dix outbreak would be the origin of a pandemic. He noted that an influenza epidemic began like a cloudburst in the population in which it first makes its appearance, for example, in a cluster of schoolchildren, as was the case with Asian flu in 1958.

Predictably, meetings of the experts were called, and a general sense of alarm prevailed, as well as a sense that something must be done to prevent an epidemic that might be a replay of 1918. All agreed that we needed to enhance national and worldwide surveillance to determine the extent of a possible major outbreak of this virus, but other courses of action were more hotly debated. Flu vaccines became available in 1944, and the primary question facing us was whether we should quickly prepare a vaccine with the Fort Dix swine flu virus strain and immunize as much of the population as possible.

In January, and for the next 10 months, David Sencer, director of CDC, frequently consulted with Harry Meyer, director of the Bureau of Biologics, and myself. Also involved in the discussions were Theodore Cooper, assistant secretary for the Department of Health, Education, and Welfare; Hope Hopps, Bureau of Biologics; Walter Dowdle, chief of the virology section at CDC; and John Seal, deputy director of NIAID. William Jordan and John LaMontagne later joined the NIAID circle. Maurice Hilleman of Merck frequently joined an informal group for intense discussions on clinical trials that were conducted in the spring of 1976 with the vaccines that had been quickly prepared by the industry.

Throughout the spring and summer, we monitored carefully for swine flu elsewhere in the world, particularly in the Southern Hemisphere, where it was winter. We received only scattered reports of an occasional case of swine flu in farmers in the Midwest, and controversy raged as to what the next steps should be. Should the vaccine be stockpiled? The argument against stockpiling was strong: the vaccine had to be given before the potential epidemic occurred in September and October, and we were racing against time. Initially, Albert Sabin insisted the vaccine should be given to children when school began in September 1976. Yet some experts preferred a "wait and see" approach.

After much consultation and discussion at the highest levels of the US government, the Public Health Service launched a program to immunize 50 million people. Following the largest voluntary mass vaccination campaign since the mass vaccination programs with Salk and Sabin polio vaccines, nearly 25% of the US population, or 45 million persons, were vaccinated by October, 10 short months after the alarm was sounded.

The epidemic, however, did not occur. The Fort Dix outbreak was a false alarm, and the American public and much of the scientific community accused us of overreacting. As someone noted, 1976 was the first time we had been blamed for an epidemic that did not take place.

Donald Burke and his group at the Johns Hopkins School of Public Health have recently calculated the basic reproductive rate (*R*_0_) of the 1976 virus. On the basis of available historical data, they calculate an *R*_0_ of 1.1–1.2. This number suggests that swine flu would not have become a major epidemic. We did not have those calculations at the time, nor were such calculations widely used. At least *R*_0_ was >1 and not <1.

These efforts to prevent an epidemic were, in some ways, like a big "fire drill." We proved it was possible to organize a mass influenza immunization program from start to finish: identify the virus, grow up stocks, prepare and field test the vaccine, provide for indemnity, and immunize a large segment of the population, all within 10 months. We learned a great deal from that drill, and I am sure we can do better the next time. The day will come when we will again retrace this race against time.

## The Fog of Epidemics

The uncertainty that surrounds any response to a microbial outbreak, the "fog of epidemics," is analogous to the fog of war, of which historians speak (*2*).

The Fog of War: Uncertainty
Where is the enemy?
What is his strength?
What counterattack?
The Fog of Epidemics: Uncertainty
Where is the microbe?
How many; how virulent; how communicable?
What counterattack?
Perceived Miscalculations
1975 Swine flu outbreak
Response too rapid
1981 HIV/AIDS occurrence
Response too slow2

In the case of swine flu, we may have acted too soon. And in the case of AIDS, we may have acted too slowly. Read the book by Neustadt and Fineberg (*3*) for a full account of our perceived folly in regard to swine flu. For an account of the perception that from 1981 to 1984, as director of NIAID, I dithered over the onset of the HIV/AIDS epidemic, read what Shilts says about me in And the Band Played On (*4*).

I relate these personal reminiscences because many who read this article will be on the firing line when future epidemics threaten, and they may either erupt or fizzle out. You will be in a fog, and you will need to exercise the best judgment you can on the basis of available surveillance information and historical context. Roy Anderson and others have been on the firing line in the United Kingdom with bovine spongiform encephalopathy and foot-and-mouth disease. And now any number of national and international organizations and the ministries of health in many countries in Southeast Asia are on the firing line in regard to avian influenza. Should we stockpile drugs? Prepare a vaccine? Cull infected flocks? When difficult choices arise, criticism is almost certain to follow, but as Harry Truman said, "If you can't stand the heat, stay out of the kitchen."

## Original Antigenic Sin

Any narrative on the swine flu episode would be incomplete without mentioning the work of Richard Shope on the possible relationship between the putative influenza virus of 1918 and its eventful residence in pigs in Iowa, where it caused an influenzalike syndrome and where it remained as a reservoir (*5*). Whatever the merits of this argument about the cause of swine flu virus infection in adults in the 1930s, of interest here is Francis's suggestion that the swine flu antibody in humans was the result of repeated exposure to human strains, and perhaps not due to prior infection with the 1918 virus. Surely his thoughts about this matter were the genesis of the concepts expressed in On the Doctrine of Original Antigenic Sin, published in 1960 (*6*).

Francis wrote, "The antibody of childhood is largely a response to dominant antigen of the virus causing the first type A influenza infection of the lifetime. The antibody-forming mechanisms are highly conditioned by the first stimulus, so that later infections with strains of the same type successfully enhance the original antibody to maintain it at the highest level at all times in that age group. The imprint established by the original virus infection governs the antibody response thereafter. This we have called the Doctrine of the Original Antigenic Sin."

Francis died in 1969 and did not live to know the full explanations for antigenic shift through reassortment of gene segments from 2 parent viruses or antigenic drift through mutation. He surely would have been in awe, as we all are, of the molecular explanation of influenza virus variation with succeeding epidemics. And yet, even with the brilliant work of Taubenberger delineating the 1918 virus (*7*), we can still ask Francis's question: Which strain will cause the next pandemic? Francis would have been cautious, but he certainly would have agreed that knowing the genetics of the 1918 virus will guide our strategy to confront future influenza pandemics. And I believe he would be cautious about the pandemic potential of the current avian influenza virus. He would warn us to keep alert to the unexpected, to be prepared for a "newe acquayantance."

## Footnotes

2 Reprinted from Emerging Infections, Richard M. Krause, Introduction, pages 1–22, Copyright 1998, with permission from Elsevier.
